# The Effects of Pre-Sleep Learning on Sleep Continuity, Stability, and Organization in Elderly Individuals

**DOI:** 10.3389/fneur.2012.00109

**Published:** 2012-07-11

**Authors:** F. Conte, G. Carobbi, B. M. Errico, G. Ficca

**Affiliations:** ^1^Department of Psychology, University of Naples IICaserta, Italy

**Keywords:** aging, arousals, learning, memory consolidation, NREM-REM cycles, sleep continuity, sleep organization, sleep stability

## Abstract

Several studies have consistently shown that pre-sleep learning is associated to changes of sleep structure. Whereas previous research has mainly focused on sleep states, namely REM and NREM amount, very little attention has been paid to the hypothesis that pre-sleep learning might improve sleep continuity, stability, and cyclic organization, which are often impaired in aging. Thus, aim of this research was to assess, in a sample of 18 healthy elderly subjects, whether a memory task administered at bedtime would determine changes in any sleep parameter, with special regard to sleep continuity, stability, and organization. To this purpose, a baseline sleep (BL), i.e., a normal sleep with 9-h time in bed (TIB), was compared to a post-training sleep (TR), with the same TIB but preceded by an intensive training session. For the latter, a verbal declarative task was used, consisting in learning paired-word lists, rehearsed, and recalled for three times in a row. To control for individual learning abilities, subjects were administered several sets of lists with increasing difficulty, until they reached an error rate ≥20% at third recall. Relative to BL, TR shows a significant reduction in the frequency of brief awakenings, arousals, state transitions, “functional uncertainty” (FU) periods, and in the percentage of time in FU over total sleep time (TST). A significant increase in the number of complete cycles, total cycle time (TCT), and TCT/TST proportion was also found. All these changes are evenly distributed over the sleep episode. No sleep stage measure display significant changes, apart from a slight reduction in the percentage of Stage 1. Scores at retest are negatively correlated with both the frequency of arousals and of state transitions. Our data suggest that pre-sleep learning can yield a beneficial re-organizing effect on elderlies’ sleep quality. The inverse correlation between recall scores and the measures of sleep continuity and stability provides further support to the role of these features in memory processes.

## Introduction

As many as 50% of older individuals complain about sleep problems, often reporting disturbed or “light” sleep, frequent night awakenings, early morning awakenings, and undesired daytime sleepiness (Foley et al., 1999; Vitiello et al., 2004). Though one relevant cause of these impairments is the presence of illness or the use of sleep-disturbing drugs, even carefully screened older adults who do not complain of sleep disturbances and with minimal medical burden show similar changes when compared to young adults (Vitiello, 2006).

Among the major objectively measured sleep changes associated to the aging process (for exhaustive reviews, see Bliwise, 1996; Ancoli-Israel et al., 2008), there are significant impairments of: (a) sleep continuity, with increased number and duration of intra-sleep awakenings (Åkerstedt et al., 2002); (b) sleep organization, expressed by a reduction of cycles’ number and a decrease of time spent in cycles over total sleep time (Salzarulo et al., 1998).

In light of the classical two-process model of sleep regulation (Borbély, 1982), the main age-related sleep changes have been explained with either a disruption of the circadian pacemaker (Dijk et al., 1999), or with age-dependent intrinsic lightening of sleep homeostatic processes (Wauquier and van Sweden, 1992). Also, it has been sometimes pointed out (Vitiello, 2006) that sleep-wake rhythms in elderly people could be altered by a number of dramatic changes in lifestyle (i.e., retirement, bereavement, and institutionalization); these, on their turn, could act via a reduction of cognitive activity, whose effects on sleep features appear to have been largely underestimated.

The idea that sleep is modulated not only by the duration of wakefulness but also by its “intensity,” resulting from physical and cognitive activity and measured through cerebral metabolic rate, was originally put forward by Feinberg (1974) to explain the “restorative role” of SWS.

In line with this suggestion, we have recently proposed (Conte and Ficca, 2012) that a specific aspect of waking activity, i.e., the learning processes, should be considered as a major additional factor in sleep regulation. In fact, a fecund line of research has consistently shown that post-training sleep (TR) displays relevant changes, both in animals and humans (see Peigneux et al., 2001 for a thorough review). These changes have usually been explained with the assumption that, following intensive learning, the sleep episode will tend to express all those components necessary for sleep-dependent memory consolidation. However, most studies have focused on post-training rebounds of quantitative sleep parameters (e.g., NREM and REM amounts, spindle density). Their controversial results, and the quite complicated picture deriving from them, have been described and commented in previous reviews of ours (Ficca and Salzarulo, 2004; Conte and Ficca, 2012). Instead, the possibility that pre-sleep learning could benefit sleep quality, in terms of a higher degree of sleep continuity, stability, and organization, has been almost totally neglected. This is somehow surprising, since there is growing evidence on the role of these parameters for the effectiveness of sleep-dependent consolidation processes.

Animal studies suggest that sleep fragmentation might disrupt memory consolidation by interrupting the natural development of biological mechanisms required for learning. Tartar et al. (2006), showed, in rats, that experimental interruptions of sleep impair spatial learning by inhibiting hippocampal long term potentiation. In mice, after the learning phase of an object recognition task, Rolls et al. (2011) used optogenetics to activate hypocretin/orexin neurons, which play a key role in arousal processes, in order to fragment sleep without affecting sleep overall amount or sleep depth. The authors found a significant decrease of performance on the subsequent day, concluding that, regardless of the total amount of sleep, a minimal unit of uninterrupted sleep is crucial for memory consolidation.

As for humans, in a sample of patients with post-traumatic stress disorder (PTSD), who often complain of sleep disturbances and memory deficits, the number of night awakenings, together with GH secretion, turned out to be an independent predictor of post-sleep recall at a word lists task (van Liempt et al., 2011).

Relatively close to the notion of “sleep continuity” is the one of “sleep stability.” If sleep-dependent memory consolidation unfolds over a continuous time course, its disruption might result not only from full behavioral awakenings, but also from any event reversing the natural build up of the sleep episode (supposed to be made by gradual deepenings periodically punctuated by transitions to REM sleep).

To our knowledge, the only attempts to provide an operational definition of sleep stability come from the bulk of studies on the cyclic alternating pattern (CAP) (Terzano et al., 2001; Parrino et al., 2011). CAP is a periodic EEG activity of NREM sleep characterized by repeated spontaneous sequences of transient events (phase A) which clearly stand out from the background rhythm (phase B) of the ongoing sleep stage, with an abrupt frequency/amplitude variation. Parrino et al. (2011) propose that specific configurations of this pattern could represent a marker of sleep instability. For instance, the prevalence in pre-school children (Bruni et al., 2005) and elderly subjects (Parrino et al., 1998) of desynchronized CAP phases (A2 subtype) over synchronized patterns (A1 subtype) would suggest a greater sleep instability in these age groups. Interestingly, A1 pattern displayed a significant increase in a post-training night, compared to baseline (Ferri et al., 2008), as well as a positive correlation with morning retest performance (Ferri et al., 2008; Aricò et al., 2010). These results provide interesting evidence on the role of slow-wave activity for learning and suggest the importance of sleep stability in memory processing.

Besides CAP as a crucial microstructural facet, here we would like to extend the definition of “stability” by considering sleep phenomena with a wider temporal resolution and not limited to patterns of NREM sleep. In fact, in our view, instability indexes should also include arousals and state transitions, which refer to a notion conceptualized by Salzarulo et al. (1997) as “functional uncertainty,” and defined as “the inability of the Central Nervous System to sustain a stable condition.” In other terms, unstable sleep phases are those in which the characteristics of one well-defined state occur only for short intervals, so that the individual appears to oscillate continuously between different states, being unable to “decide” for one or another. This kind of sleep pattern would be particularly evident in extreme age groups (children and elderly individuals), as well as in those subjects affected by fragmenting sleep disorders.

Finally, the hypothesis of a role of sleep cycles for learning processes has been underlined, together with the behavioral, biological, neurophysiological, and neuroanatomical evidence supporting it, in the frame of what we call “sleep organization” (Ficca and Salzarulo, 2004). Experimentally, this hypothesis was confirmed by a few findings. In a sample of healthy elderly individuals, Mazzoni et al. (1999) found that post-sleep recall of word pairs was significantly correlated with average duration of NREM REM cycles and with the proportion of time spent in cycles total cycle time (TCT) over total sleep time (TST), whereas it was not related to any other sleep measure. One year later, it was shown that the experimental disruption of sleep cycles determined significantly worse recall of word pairs, relative both to undisturbed sleep and to fragmented sleep with sleep cycle preservation (Ficca et al., 2000). Finally, the number of sleep cycles was significantly correlated with performance at a visuo-spatial task in a group of patients with chronic non-restorative sleep (Göder et al., 2007).

Due to the aforementioned traits of discontinuity, instability, and disorganization of elderlies’ sleep, a “re-compacting” and “re-organizing” effect of pre-sleep training would be particularly beneficial for this population.

Therefore, the aim of this study is to assess whether a learning task administered before sleep onset determines changes in any sleep parameter of the subsequent sleep episode, with special regard to sleep continuity, stability, and organization, in a sample of healthy elderly subjects.

## Materials and Methods

### Subjects

Prior to subjects’ recruitment, the design of the study was submitted to the Ethical Committee of the School of Psychology, University of Naples II, that approved the research and certified that the use of human subjects was performed according to acceptable standards.

Eighteen elderly volunteers (F = 10, M = 8; age range: 65–85 years, mean age: 72.5 ± 5 years), all home-dwelling, were recruited from the general population.

Participants were screened through: (a) a brief *ad hoc* interview to collect general demographic data and information on medical condition and health habits; (b) the Mini Mental State Examination (MMSE), a brief 30-point questionnaire test, commonly used in the clinical context to screen for cognitive impairment (Folstein et al., 1975); (c) the Pittsburgh Sleep Quality Index (PSQI), a questionnaire on sleep habits and quality (Buysse et al., 1989).

Inclusion criteria were the following: age ≥65 years; absence of any relevant somatic disease, either acute or chronic; no evidence of current or past psychiatric illness; absence of cognitive decline (MMSE score ≥25); no symptoms of sleep apnea or other respiratory disorders; no complaints of insomnia or daytime sleepiness; absence of relevant sleep-wake rhythm disruptions; no history of drug or alcohol abuse; being a non-smoker; limited caffeine and alcohol consumption (no more than three cups of coffee and a glass of alcohol a day).

### Procedure

Each subject underwent three nights of sleep recording at home, separated by an interval of 3 days to 1 week. An adaptation night was followed, in balanced order, by two experimental conditions: (a) baseline sleep (BL), i.e., a normal night’s sleep with 9-h time in bed (TIB) allotted, (b) Post-TR, with the same maximum TIB but preceded by a training session.

For the 3 days preceding each sleep recording session, subjects were requested to maintain regular bed- and rise-times and regular napping habits. Specifically, in order to assure the maintenance of *habitual* sleep-wake rhythms, subjects were allowed to take naps only if these represented a daily habit. In that case, naps should not differ from the subject’s usual naps neither in length nor in circadian placement. To control for these variables, participants were asked to complete a sleep log for a week preceding each sleep recording night and to wear an actimeter from 9 a.m. of each recording session day until 9 a.m. of the following morning. Furthermore, during the days scheduled for recording, participants were requested to avoid cognitively engaging activities (such as reading, solving crossword puzzles, playing cards, etc.).

On TR day, the experimenter arrived at the subject’s house approximately 1 h before usual bedtime and proceeded to electrode montage. The subject was then administered the training session and was instructed to go to bed immediately after that. In the BL condition, instead, the subjects went to bed immediately after electrode montage. The following morning, subjects completed the sleep diary and, in the TR condition, retest was performed 30 min after awakening.

To control for mood and sleepiness levels, a Visual Analog Scale for mood (VAS-mood, McCormack et al., 1988) and the Karolinska Sleepiness Scale (KSS, Akerstedt and Gillberg, 1990) were administered immediately before bedtime on the recording night and at awakening the following morning. In addition, in the TR condition, the two scales were also completed in the evening before training administration and immediately before retest in the morning.

### Polygraphic and actigraphic recordings

Polysomnographic (PSG) recording was performed in accordance with standardized techniques (Rechtschaffen and Kales, 1968), using digital EEG, EMG, and EOG signals acquired through a multi-channel ambulatory recorder (XLTEK Trex™ HD Home Sleep).

For actigraphic monitoring, a Micromini Motionlogger Actimeter was used. The actimeter was worn on the wrist of the non-dominant hand.

### Learning task

For the training session, a verbal declarative task, consisting in learning paired-word lists, was developed *ad hoc* for the experiment. Lists were made up of bysillabic concrete nouns, and they were balanced for number of semantically associated pairs and mean frequency of use of words (Bortolini et al., 1971). The total number of word pairs was 168, divided as follows: 3 lists of 8 word pairs, 3 lists of 12, 3 lists of 16, and 3 lists of 20.

At the beginning of the training session, each subject was informed that a recall test would be administered the following morning. During list presentation, the experimenter read each word pair out loud, with an interval of 1 s between each pair. Immediately after list presentation, the experimenter read only the first member of each pair and the subject was asked to recall the second one. In order for learning to take place, this procedure was repeated three times for each list.

**Figure 1:**
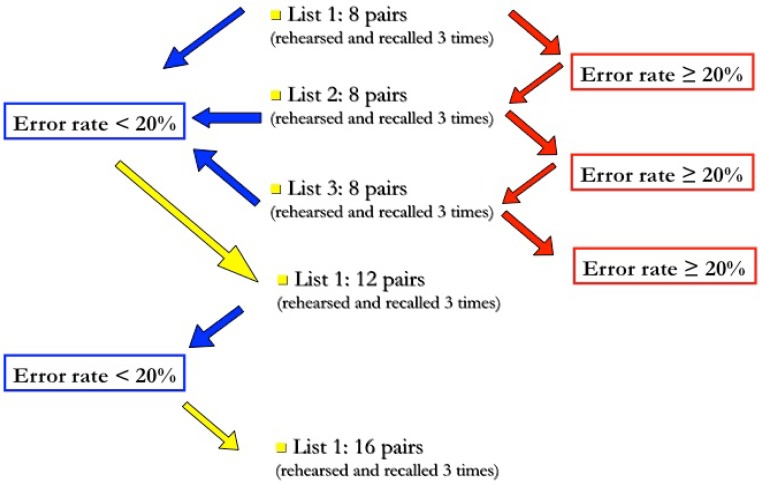
**Task administration procedure**. The trial starts with the administration of a 8-pairs list; if, at third recall, the number of errors is lower than 20%, the subject will proceed to the next difficulty level, i.e., a 12-pairs list, otherwise he will continue with another list of the same length. The trial is terminated when the subject does not succeed in decreasing his error rate below 20% at third recall; otherwise, this same procedure is repeated with longer (12-, 16-, and 20-pairs) lists.

To control for individual learning abilities and amount of cognitive effort, each subject was presented several sets of lists with increasing length, until they reached an error rate of 20% at third recall (see Figure 1 for scheme and explanation).

At retest in the morning, subjects were presented only the first members of the pairs belonging to the lists administered to that subject the night before. For data analysis, scores at retest were calculated as percentage of words recalled in the morning over the total number of words recalled by the subject in the training session.

### Sleep measures

Sleep recordings were scored through visual inspection, according to standard criteria (Iber et al., 2007). However, as in Mazzoni et al. (1999), SWS was scored without considering the amplitude criterion, following Webb and Dreblow’s (1982) criteria.

Sleep scoring was performed by an expert technician who was blind to the condition to which the recording belonged. To verify the reliability of scoring, randomly selected sleep recordings were also scored by another expert technician. Inter-rater agreement was 95.2%.

Classical sleep quantitative variables considered in our study were: TIB, TST (total amount of time, expressed in minutes, from the first appearance of Stage 1 sleep to morning final awakening), actual sleep time (AST, i.e., total amount of time spent in sleep, expressed in minutes), sleep latency, sleep efficiency, sleep stage proportions, percentage of wake after sleep onset (WASO) over TST.

An additional set of parameters was used to specifically address sleep continuity, stability, and organization.

As for sleep continuity: (a) number of awakenings <2 min per hour of AST; (b) number of awakenings ≥2 min per hour of AST; (c) number of awakenings from Stage 1, Stage 2, SWS, REM sleep per minute of that stage; (d) mean duration of awakenings; (e) number of arousals per hour of AST (arousals were defined as all transitions to shallower NREM sleep stages and from REM sleep to stage 1); (f) number of arousals from stage 2, SWS, REM sleep per minute of that stage.

Concerning sleep stability: (a) number of “state transitions” per hour of TST (“state transitions” were defined as all transitions from one state to another, including all those to and from wake, and all those from one stage to another); (b) number of “functional uncertainty periods” (FU periods) per hour of TST (FU periods were defined as periods in which a minimum of three state transitions follow one another with no longer than 1 min and a half intervals); (c) mean duration of FU periods; (d) percentage of total time spent in FU (TFU) over TST.

Finally, with respect to sleep organization: (a) number of complete sleep cycles, defined as sequences of NREM and REM sleep (each lasting at least 10 min), not interrupted by periods of wake and/or stage 1 longer than 2 min; (b) TCT, i.e., total time spent in cycles (minutes); (c) percentage of TCT over AST; (d) cycle mean duration.

### Statistical analysis

Due to non-normal distribution of variables, non-parametric statistics was chosen for data analysis. BL and TR variables were compared by means of Wilcoxon’s signed rank test (for directed hypothesis).

Spearman’s analysis of correlation was carried to assess the association between TR sleep parameters and recall scores at morning awakening.

Finally, TST was divided in quarters for each sleep episode and a two-way analysis of variance (ANOVA), with “condition” and “quarter” as factors, was carried to explore the time course of the effects of training on sleep continuity and stability variables across the sleep episode.

## Results

Two subjects had to be excluded from analyses due to the absence of fragmentation in their BL sleep (0 awakenings in both subjects). Thus the final sample for data analysis included 16 participants (F = 9, M = 7; age range: 65–85 years, mean age: 70.1 ± 6.6 years).

### Quantitative sleep variables

A significant increase of AST emerged at TR, compared to BL. Reduced WASO was also found in TR vs. BL, paralleled by a significant increase of sleep efficiency. No significant differences were observed between the two conditions in stage amount percentages, with the only exception of a slight reduction of Stage 1 in TR (Table 1).

**Table 1:** **Quantitative sleep variables**.

	Baseline	Training	Wilcoxon’s *z*	*p*-Value
Time in bed (min)	406.2 ± 88.7	410.8 ± 44.9	0.099	ns
Total sleep time (min)	391.4 ± 88.6	398.2 ± 51.9	0.356	ns
Actual sleep time (min)	326.7 ± 99.9	360.5 ± 58.6	1.689	**0.04**
Latency (min)	14.7 ± 13.9	12.6 ± 11.8	0.296	ns
Stage 1 (%)	21.2 ± 8.8	15.7 ± 9.5	1.689	**0.04**
Stage 2 (%)	44.5 ± 15.2	39.9 ± 8.4	1.334	ns
SWS (%)	12.9 ± 9.6	17.8 ± 10.2	1.423	ns
REM (%)	16.4 ± 10	20.4 ± 5.7	1.511	ns
WASO (%)	17.3 ± 13.8	10.8 ± 5.6	2.223	**0.01**
Sleep efficiency	79.9 ± 15.1	86.7 ± 7.6	2.312	**0.01**

*Bold represents the significant p-values*.

No other quantitative sleep variable showed significant differences between BL and TR in (Table 1).

### Sleep continuity

**Figure 2:**
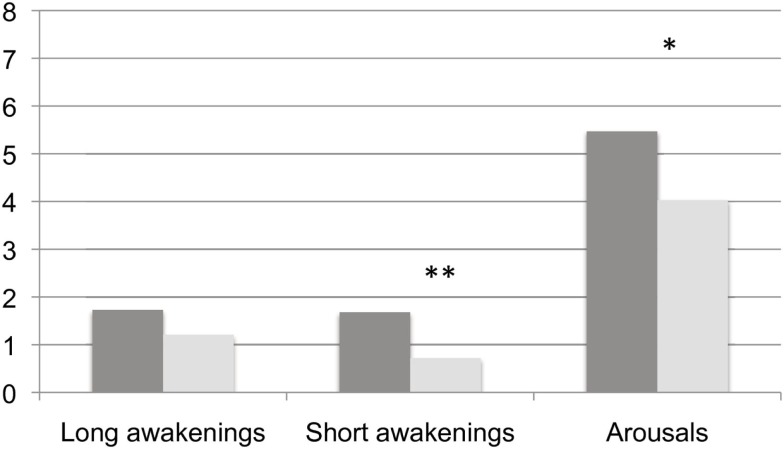
**Frequency of behavioral awakenings and arousals in the two experimental conditions**. Dark gray columns: BL, light gray columns: TR Frequencies of awakenings and arousals are calculated over hours of AST. *, ≤0.05 and **, ≤0.01.

We have summarized the comparisons of continuity variables between BL and TR in Figure 2. Though no modifications were found in the frequency of long – i.e., lasting 2 min or more – awakenings (BL: 1.73 ± 1.53 vs. TR: 1.21 ± 0.54, Wilcoxon’s *z* = 1.245, ns), we detected a significant decrease at TR in the frequency of both short – i.e., less than 2 min – awakenings (BL: 1.68 ± 1.01 vs. TR: 0.72 ± 0.74, Wilcoxon’s *z* = 2.223, *p* = 0.01) and arousals (BL: 5.47 ± 2.18 vs. TR: 4.29 ± 0.92, Wilcoxon’s *z* = 1.689, *p* = 0.04). Mean awakenings duration, not displayed in the Figure, was not modified by training (Wilcoxon’s *z* = 0.140; ns).

Table 2 shows the distribution of fragmenting events in the different sleep stages.

**Table 2:** **Sleep fragmentation in different sleep stages**.

	Baseline	Training	Wilcoxon’s *z*	*p*-Value
AWAKENINGS FREQUENCY*				
from Stage 1	0.13 ± 0.09	0.08 ± 0.05	1.067	ns
from Stage 2	0.05 ± 0.07	0.03 ± 0.02	0.089	ns
from SWS	0.04 ± 0.06	0.03 ± 0.05	0.889	ns
from REM	0.05 ± 0.05	0.03 ± 0.05	1.423	ns
AROUSALS FREQUENCY*				
Stage 2 to Stage 1	0.13 ± 0.07	0.08 ± 0.04	1.689	**0.04**
SWS to Stage 2	0.39 ± 0.23	0.27 ± 0.22	1.956	**0.02**
SWS to Stage 1	0.03 ± 0.05	0.01 ± 0.01	1.820	**0.03**
REM to Stage 1	0.02 ± 0.01	0.02 ± 0.03	0.178	ns

*Awakenings and arousals from a certain stage are calculated as frequencies over the total time spent in that stage (minutes).

*Bold represents the significant p-values*.

No awakening measure displayed significant changes at TR relative to BL.

As for arousals, a significant reduction in the frequency of arousals from Stage 2 to Stage 1, from SWS to Stage 2 and from SWS to Stage 1 was found at TR, whereas those from REM sleep to Stage 1 were unmodified.

### Sleep stability

A highly significant reduction emerged at TR for the frequency of FU periods per hour of TST, and for percentage of total time spent in FU over TST. The frequency of state transitions also displayed a significant decrease at TR, while the mean duration of FU periods did not show any changes (Table 3).

**Table 3:** **Sleep stability**.

	Baseline	Training	Wilcoxon’s *z*	*p*-Value
State transitions TST (h)	15.5 ± 5.6	11.8 ± 4.0	1.956	**0.02**
FU periods TST (h)	1.2 ± 0.6	0.7 ± 0.3	1.867	**0.03**
TFU TST (%)	9.7 ± 5.6	5.5 ± 2.2	2.223	**0.01**
FU periods mean duration (min)	4.3 ± 1.5	4.4 ± 0.8	0.089	ns

*Bold represents the significant p-values*.

### Sleep organization

All sleep cycles measures (number of cycles and TCT, both absolute and relative to AST), except for cycle mean duration, showed significant increases from BL to TR (Table 4).

**Table 4:** **Sleep organization**.

	Baseline	Training	Wilcoxon’s *z*	*p*-Value
*N* cycles	0.9 ± 1	2.1 ± 1.7	2.200	**0.01**
TCT (min)	48.14 ± 56.6	113.9 ± 102.5	2.073	**0.02**
TCT AST (%)	13.1 ± 14.4	30.8 ± 24.8	1.955	**0.02**
Cycle mean duration (min)	30 ± 32.6	43.6 ± 26.1	1.007	ns

*Bold represents the significant p-values*.

### Analysis of correlation

**Figure 3:**
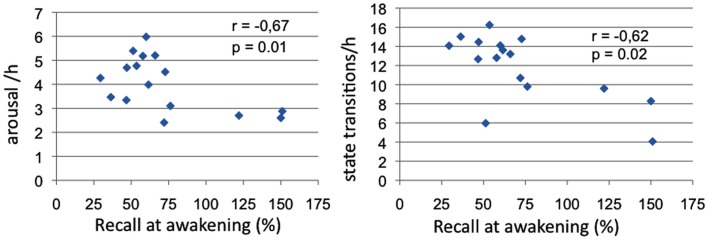
**Sleep measures correlated with memory recall at awakening**. Panel 1: Correlations of arousal frequency with recall at awakening (% over pre-sleep recall); Panel 2: Correlations of state transitions frequency with recall at awakening (% over pre-sleep recall).

Significant negative correlations between morning recall performances and TR sleep variables emerged for total arousal frequency (Spearman’s ρ = −0.67, *p* = 0.01) and state transition frequency (Spearman’s ρ = −0.62, *p* = 0.02; see Figure 3).

### Time distribution of sleep continuity and stability measures

Analysis of variance did not show a significant main effect of the factor “quarter” for any of the sleep continuity and stability measures, nor any significant interaction “condition × quarter” (Table 5).

**Table 5:** **Time distribution of sleep fragmentation**.

	Quarter *F*	Condition *F*	Quarter × condition *F*
Short awakenings AST (h)	2.154	4.717**	1.272
Long awakenings AST (h)	1.657	1.584	0.570
Arousals AST (h)	1.894	7.596*	0.336
State transitions TST (h)	0.769	4.601*	1.347
FU periods TST (h)	0.484	5.274**	0.709
TFU TST (%)	0.510	9.655**	0.354

* < 0.05 and ** < 0.01.

## Discussion

The presence in the baseline nights of a notable degree of sleep fragmentation and disorganization, expressed by the high number of awakenings and arousals and by the low sleep efficiency, as well by a low TCT/TST, proves that sleep features in our sample are consistent with what is known on elderlies’ sleep (Dijk et al., 2001; Vitiello, 2006; Ancoli-Israel et al., 2008). In fact, only 2 of the 18 recruited subjects showed no behavioral awakenings at baseline and were excluded for aberrant values, whereas, for the remainder subjects, the mean absolute number and the frequency of awakenings were even higher than those reported in most literature on aging (for a review, see Åkerstedt et al., 2002).

This evidence supports our preliminary hypothesis that the chosen sample was adequate to explore a possible re-organizing effect of the cognitive manipulation.

The main result of the present study is the significant impact of the verbal learning task administered before bedtime on the subsequent sleep episode, both in terms of sleep continuity and stability, and in terms of sleep cyclic organization.

As for the observed reduction of arousals and awakenings, the protective effect of pre-sleep training against fragmentation appears to be effective mainly on weaker arousing events, i.e., those which determine shifts to lighter sleep stages or very brief awakenings; instead, it does not seem able to prevent stronger arousing drives (those leading to full, and often very long, behavioral awakenings) which are probably the result of a sum of different factors (internal and or external).

Our analysis of the time course of sleep fragmentation proves it to be quite different in the elderly than in young subjects. While the latter are characterized by a tendency to spontaneously wake up from REM sleep, therefore with a higher degree of sleep fragmentation in the last part of the sleep episode (Langford et al., 1972; Murphy et al., 2000), and longer wake in late than in early sleep cycles (Merica and Gaillard, 1986), the former seem vulnerable to fragmentation all over the sleep episode. In the present study, this is actually true not only for behavioral awakenings, but also for arousals and state transitions. This set of data apparently gives support to what proposed some years ago: “either sleep is shallow and fragile in elderly patients, thus diminishing differences in arousability between sleep stages, or sleep is less shielded against the intrusion of external disturbances which may lead to arousals and awakening independent of the ongoing sequence of sleep stages” (Åkerstedt et al., 2002). Very interestingly, also the effect of training in reducing this high arousability seems to be evenly exerted across the whole night.

It must be acknowledged that a limitation in our study is the use of only one among the many different operational definitions of the term “arousal” (Ficca, 2002), which is aimed to detect macrostructural superficializations of sleep in the frame of the epoch-by-epoch visual EEG scoring. It would be important to replicate these findings with other definitions, such as the one adopted by American Sleep Disorder Association (1992), which refers to “EEG changes whose duration may be very short, even not longer than 3 s.”

Also the signals of sleep instability, namely the state transitions and the FU periods, that are conspicuously present in baseline nights, undergo a significant decrease in post-TR.

Our results on sleep stability variables, together with recent data from clinical studies on fibromyalgia (Kishi et al., 2010) and sleep-disordered breathing (Swihart et al., 2008), suggest the possible utility of including these measures in standard sleep assessments on both normal and clinical populations as further indexes of sleep quality. These parameters could usefully complement the evaluations of the CAP (Terzano et al., 2001), which, although limited to NREM sleep, was shown to represent a reliable marker of sleep instability (Parrino et al., 2011).

Results on sleep organization suggest that bedtime training has a positive impact also on sleep cycling, since the number of cycles and TCT, both absolute and relative to TST, were significantly increased in the TR condition. Instead, cycles mean duration was unmodified. However, this result could be explained by the very “conservative” definition of cycle adopted (see Materials and Methods), in analogy with previous studies (Mazzoni et al., 1999; Ficca et al., 2000). Rather than on already long uninterrupted cycles, it is well possible that training would have exerted its lengthening effect on those short NREM-REM sequences, which have not been included as actual sleep cycles in the analysis, since not fulfilling the required criterion of a minimum duration for both sleep states.

Overall, these findings represent encouraging evidence on the possibility to exploit planned pre-sleep training sessions to improve sleep and subsequent wake quality in all those conditions affected by fragmented and disturbed sleep.

Of course it might be argued that the changes we have observed in post-TR are specific for the aged population. However, a similar pattern of results has already been obtained, by means of the same experimental paradigm, on a sample of nine young subjects complaining of frequent awakenings and disturbed sleep (Conte et al., 2010).

Some remarks can also be made with regard to the debate on the mechanisms underlying the effect of sleep on memory consolidation.

The significant increase in sleep cycles variables is consistent with our working hypothesis, assuming, in the frame of the “sleep cycles model” (Mazzoni et al., 1999; Ficca et al., 2000), a central role of sleep organization for memory processing during sleep.

On the other hand, further exploration of this issue is required in light of the absence, in our study, of significant correlations between cycles measures and memory recall, at variance with previous data (Mazzoni et al., 1999; Göder et al., 2007).

As for sleep continuity and stability, the reduction at TR of brief awakenings, arousals, state transitions, and FU measures induces us to confirm the hypothesis that these features also play a role in sleep-dependent consolidation, in agreement with a few other studies (Tartar et al., 2006; Rolls et al., 2011; van Liempt et al., 2011). Obviously, a necessary assumption is that the observed post-TR changes are specifically dependent on the triggering of consolidation processes. The significant correlations between recall performances and continuity and stability variables, seems to us encouraging supports to this assumption.

However, the elderly population poses a specific problem to the interpretation of these results, concerning the age-related modifications of slow-wave activity. Despite some authors’ proposal (Webb and Dreblow, 1982; Lombardo et al., 1998) that the reduced delta power in the elderly depends solely on reduced amplitude of delta waves, rather than on an inability to produce them, other authors (e.g., Feinberg, 1989) claim that the elderly individuals’ brain becomes less capable to produce a visually detectable slow-wave rebound. If this is the case, then the characteristics of the sample studied do not allow us to fully exclude: (a) that the contribution of an unspecific effect (more generally dependent on cognitive “effort” and fatigue), which could determine a homeostatic SWS rebound, is at work; (b) that a shift toward more slow-wave element production, that has been connected to synaptic potentiation and memory consolidation in previous articles (e.g., Tononi and Cirelli, 2003; Huber et al., 2004), would have been detectable even in this elderly population with more fine-grained techniques of SWA analysis, as it was shown in a previous paper by Ferri et al. (2008) through measurement of CAP parameters. For these reasons, future studies could add important information by adding a non-learning performance task control condition, as well as more sensitive measures of slow-wave changes.

A final remark concerns the implications of our findings in the understanding of sleep quality disruption in aging.

It has been shown that the worsening of elderlies’ sleep quality is partly determined by changes in lifestyles and daily activities intervening with aging and particularly with retirement, such as social isolation (Ohayon et al., 2001; National Sleep Foundation Poll, 2003), or reduction of physical activities (Foley et al., 1999).

However, while this evidence has so far been attributed only to the disruption of the “zeitgebers” system, either photic (light-based) or non-photic (e.g., exercise activities, scheduled meals, or social cues; Vaz Fragoso and Gill, 2007), we propose that the impoverished socio-relational network of the elderly negatively affect his sleep quality through the consequent reduction of cognitive activity.

The analysis of our results opens the way to a series of interesting theoretical speculations to be explored in future studies:

(a) It is possible that post-TR is characterized, along with the sleep variables we have considered, by some microstructural correlates supporting its greater stability and continuity, that is a lower tendency to awakenings and superficializations. For instance, in a very recent study aimed at investigating sleep maintenance ability in young subjects, Dang-Vu et al. (2010) found that the subjects displaying higher arousal thresholds in the face of environmental sounds showed higher spindle densities. The authors concluded that, in addition to perhaps actively contributing to memory consolidation, spindles may shield sleep from disruption, allowing consolidating processes to operate unhindered.
(b) In our opinion, although we currently have experimental evidence concerning only declarative tasks, i.e., recall of paired words lists (Mazzoni et al., 1999; Ficca et al., 2000), and delayed recall of the Rey–Osterreith Complex Figure task (Göder et al., 2007), there is no particular reason to rule out a contribution of “good sleep quality” (in terms of adequate cycling, continuity, and stability), to the consolidation of different types of tasks usually grouped under procedural learning.

In conclusion, the findings of our study support the hypothesis that administration of learning tasks may improve subsequent sleep quality in the elderly individuals, in terms of continuity, stability, and organization. The promising applicative implication is the possibility to plan specific training sessions in populations affected by disturbed sleep, both in natural (aged individuals) and clinical settings (pathologies fragmenting sleep).

Furthermore, our results encourage to conceive further studies on the role of sleep continuity, stability, and organization for sleep-dependent memory consolidation.

## Conflict of Interest Statement

The authors declare that the research was conducted in the absence of any commercial or financial relationships that could be construed as a potential conflict of interest.
